# Comparison of the Cannabinoid and Terpene Profiles in Commercial Cannabis from Natural and Artificial Cultivation

**DOI:** 10.3390/molecules28020833

**Published:** 2023-01-13

**Authors:** Fereshteh Zandkarimi, John Decatur, John Casali, Tina Gordon, Christine Skibola, Colin Nuckolls

**Affiliations:** 1 Department of Chemistry, Columbia University, New York, NY 10027, USA; 2 Huckleberry Hill Farms, 9415 Briceland Rd, Garberville, CA 95542, USA; 3 Moonmade Farms, P.O. Box 5, Garberville, CA 95542, USA; 4 Cosmic View, P.O. Box 1255, Novato, CA 94948, USA

**Keywords:** cannabinoids, terpenes, LC-MS, GC-MS, commercial cannabis

## Abstract

Interest in cultivating cannabis for medical and recreational purposes is increasing due to a dramatic shift in cannabis legislation worldwide. Therefore, a comprehensive understanding of the composition of secondary metabolites, cannabinoids, and terpenes grown in different environmental conditions is of primary importance for the medical and recreational use of cannabis. We compared the terpene and cannabinoid profiles using gas/liquid chromatography and mass spectrometry for commercial cannabis from genetically identical plants grown indoors using artificial light and artificially grown media or outdoors grown in living soil and natural sunlight. By analyzing the cannabinoids, we found significant variations in the metabolomic profile of cannabis for the different environments. Overall, for both cultivars, there were significantly greater oxidized and degraded cannabinoids in the indoor-grown samples. Moreover, the outdoor-grown samples had significantly more unusual cannabinoids, such as C4- and C6-THCA. There were also significant differences in the terpene profiles between indoor- and outdoor-grown cannabis. The outdoor samples had a greater preponderance of sesquiterpenes including β-caryophyllene, α-humulene, α-bergamotene, α-guaiene, and germacrene B relative to the indoor samples.

## 1. Introduction

Until recently, cultivation and use of cannabis plants for medicinal, industrial, and recreational use were strictly prohibited and there is severely limited scientific research in this field [1,2]. However, due to recent shifts toward legalizing cannabis use in many locations, understanding its chemical diversity is of great importance for consumers and producers of cannabis. The bioactive properties of cannabis are derived from the plethora of secondary metabolites, which include cannabinoids, terpenoids, sterols, and flavonoids. Each of them has been identified and described across cannabis inflorescences, leaves, stem barks, and roots [3,4,5,6]. The chemical profile of particular metabolites has mainly been studied as a function of the plant’s genetics and environment. It stands to reason that the physiological effects and therapeutic benefit of different cannabis strains is linked to the diversity and the quantities of these secondary metabolites [7,8].

A common method in cannabis cultivation to avoid genetic variations is to grow genetically identical plants from clones. Moreover, by implementing biotechnological tools such as genetic engineering, it is possible to produce plants with overexpressed genes responsible for the biosynthesis of particular bioactive metabolites [7,9]. Environmental conditions such as mineral nutrition, temperature, humidity, soil bacteria, and light intensity/spectra are important factors affecting the chemical composition and secondary metabolism in cannabis plants [10,11,12,13]. The optimal mineral nutrients such as nitrogen (N), phosphorus (P), calcium (Ca), iron (Fe), and potassium (K) are essential for both the vegetation and flowering development stage of cannabis production [14,15,16,17,18,19]. For example, cannabis supplementation with nitrogen has been suggested for maximal inflorescence biomass production, while an increase in nitrogen supply may cause depletion in the levels of major cannabinoids such as Δ9-tetrahydrocannabinolic acid (THCA) and cannabidiolic acid (CBDA) [14]. THCA and CBDA are synthesized from cannabigerolic acid (CBGA), a common precursor, by THCA and CBDA synthase, respectively [20]. Subsequently, they can be decarboxylated through various processes, such as heating, light exposure, or chemical reactions into CBD and THC. It has been reported that phosphorus-enhanced fertilizer increases the levels of CBD, CBG, and cannabinol (CBN), while decreasing THC [15]. Interestingly, the concomitant N, P, and K supplementation can cause accumulation of CBG in flowers and depletion in the level of CBN/CBNA in both flowers and inflorescence leaves [15]. Moreover, temperature, relative air humidity, and CO_2_ concentrations are other abiotic factors influencing cannabinoid biosynthesis [11]. Significant variations in the cannabis plant morphology and secondary metabolism have also been reported in response to light intensity and quality [21,22,23,24].

Cannabis can be cultivated in open fields (outdoors), in greenhouses under a protected environment, or in controlled growing spaces (indoors). Outdoor cultivation exploits Mother Nature for light, temperature, and humidity, while indoor cultivation is energy-consuming and costly, mainly due to air conditioning, artificial lighting, heating, and ventilation [25]. The vast majority of recent studies have been applied to fulfill the demand for optimal efficiency of the controlled growing system and yield maximization of cannabis in indoor conditions; therefore, there is limited information on the effect of outdoor cultivation on the quantity and variability of the secondary metabolites. During the period of prohibition, it was difficult to grow cannabis outdoors under optimized conditions, and as such, comparisons of outdoor- and indoor-grown cannabis are lacking in the scientific literature. This study compares the metabolic profile of commercial cannabis from two different cultivars, grown indoors using artificial light and artificially grown media, with samples having identical genetics but grown outdoors in living soil with sunlight. We found that in general, the commercial samples that were sun-grown (naturally) have less oxidized and degraded cannabinoids and more terpenes (quantity and type of terpenes), particularly the sesquiterpenes, than the genetically identical commercial samples grown indoors, under artificial lights utilizing artificial growth media.

## 2. Results and Discussion

Cannabis is an annual plant that can be grown efficiently indoors under controlled conditions or outdoors under full spectrum sunlight [11]. Secondary metabolism in cannabis plants is influenced by several environmental cultivation conditions. To date, the effects of outdoor cultivation factors compared to indoor conditions on the cannabinoid and terpene profiles in cannabis have not been fully studied.

During inflorescence, the cannabis plant produces a plethora of cannabinoids and terpenes in the glandular trichome cells [26]. It is staggering and remarkable the number of these molecules that the plant produces. There is also added complexity that the cannabinoids can be oxidized in a multitude of ways. This creates two types of cannabinoids. The first are ones that are intrinsic to the cannabis plant because they are made by a biological pathway in the plant [27]. We refer to these as intrinsic cannabinoids. There are also other cannabinoids that are extrinsic to the cannabis plant that are created through subsequent reactions due to their environment, such as oxidation or photochemistry. We refer to these as extrinsic cannabinoids. The terpenes broadly fall into four main categories: monoterpenes (10 carbon), monoterpenoids (oxygenated terpenes), sesquiterpenes (15 carbon), and sesquiterpenoids (oxygenated sesquiterpenes) [27].

In this study, we used commercial cannabis samples that are cloned from a common parent but which are grown both indoor and outdoor under optimized conditions. The outdoor samples were grown in raised beds using a proprietary mixture of all-natural soil and composts under full sunlight. The indoor samples were grown under artificial light in a proprietary growth medium. The outdoor samples were stickier to the touch and were much more pungent than the indoor samples. The morphology and color of the flowers were similar. Each of the samples was from the same season to eliminate issues of large differences in age between the samples. Therefore, we can assess the importance of the two environments on the terpenes and cannabinoids metabolite compositions in two cultivars.

### 2.1. Principle Component Analysis of Cannabinoids

**Figure 1:**
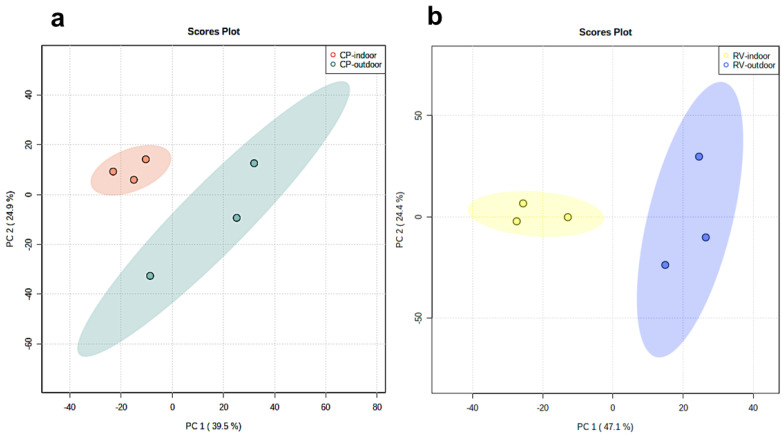
Principal component analysis (PCA) score plots of the extracted metabolic features obtained by untargeted LC-MS analysis in the negative ionization mode (**a**) CP-indoor (red color) and CP-outdoor (green color) and (**b**) RV-indoor (yellow color) and RV-outdoor (blue color).

We performed an untargeted LC-MS-based metabolomic to analyze the metabolic profile of two cultivars, CP and RV, each genetically identical produced through clones, grown either indoors or outdoors under optimized conditions (Supplementary Figure S1a). We analyzed three samples of each (n = 3) for both indoor and outdoor samples. The untargeted LC-MS analysis of the samples resulted in the detection of 1001 and 1316 features in the positive and negative ESI modes, respectively. Unsupervised principal component analysis (PCA) of the extracted features showed tight clustering of QC samples and clear serrations of indoor versus outdoor groups (Figure 1 and Supplementary Figure S1b). The tight clustering of the multiple injections of the QC sample implies no drift over time and confirms the reproducibility of the LC-MS system. We further examined the data to ensure that no terpenes or flavonoids were present in this analysis. The PCA score plots in the negative mode indicate the differences in the cannabinoid profiles (Figure 1). The PC1 (describing the variation between groups) from the two plots was found to be 39.5% and 47.1%, and PC2 (which describes the variation within the groups) was 24.9% and 24.4% for the CP and RV models, respectively. Thus, the PCA score plots represents a distinctive clustering due to metabolic differences. Supplementary Figure S2a,c show the loadings plots, displaying the discriminative ion features localized in the peripheral (extreme values in PC1 and PC2) areas of the plots between indoor-grown and outdoor grown cultivars.

Although being genetically identical, the indoor and outdoor samples from either of these two cultivars are completely distinguished by the composition of their cannabinoids. While we are applying this analysis here to different methods of cultivation, this type of analysis of the cannabinoids will enhance our understanding of the effects the environment and genetics have on the cannabinoids produced and also in the understanding of how to classify the multitude of different cultivars of cannabis that are commercially available.

### 2.2. Cannabinoid Analysis

**Figure 2:**
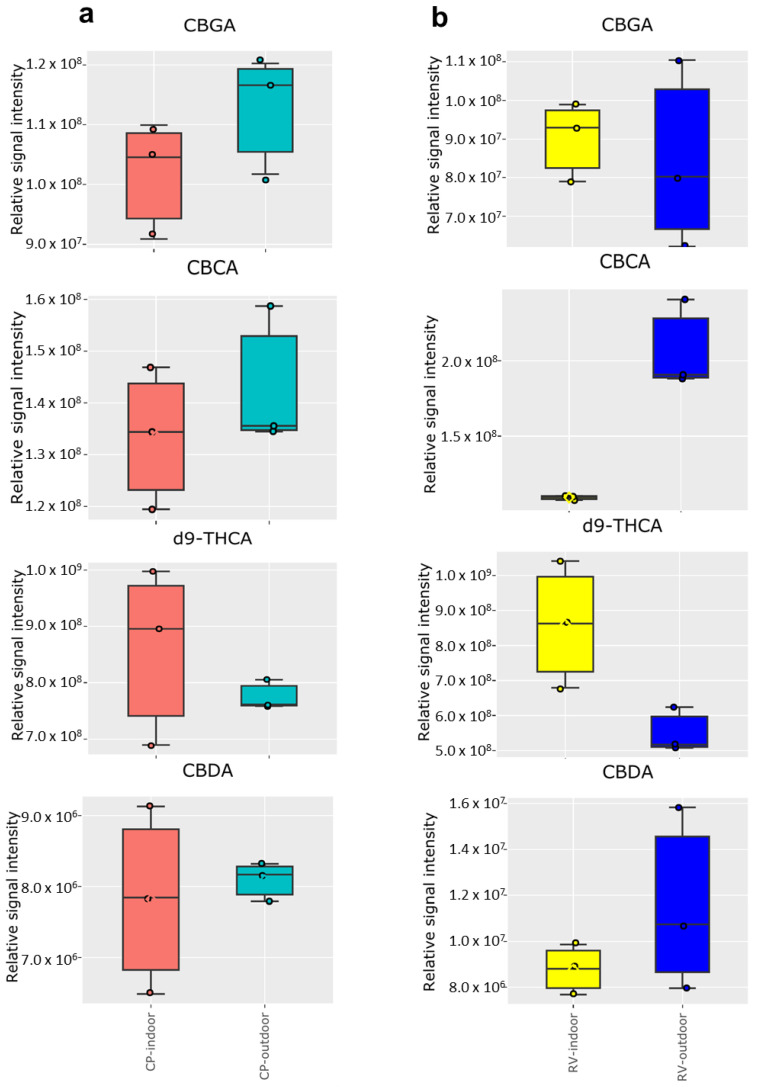
Comparison of the primary, intrinsic cannabinoids, CBGA, CBCA, Δ^9^-THCA, and CBDA detected by LC-MS analysis in (**a**) CP-indoor and CP-outdoor; (**b**) RV-indoor and RV-outdoor (n = 3 independent samples per group).

To further understand the differences between the molecular profiles that gave rise to the disparate PCA results when comparing indoor versus outdoor cultivation, we conducted targeted cannabinoid analysis of the primary, intrinsic cannabinoids, CBGA, CBCA, Δ^9^-THCA, and CBDA for CP (Figure 2a) and RV (Figure 2b).

We found little difference between the indoor- and outdoor-grown samples for these primary cannabinoids except CBCA and Δ^9^-THCA, which are enhanced and depleted significantly in the RV-outdoor samples, respectively. This series is of importance because CBGA is a common precursor to CBCA, Δ^9^-THCA, and CBDA that occur via three different biochemical pathways by particular synthases, among which the most prominent are tetrahydrocannabinolic acid-, cannabidiolic acid-, and cannabichromenic acid-synthase, leading to the production of THCA, CBDA, and CBCA, respectively [28,29]. IN addition, then they can be decarboxylated through various processes such as light exposure, heating, or through chemical reactions [30].

The corresponding data for the decarboxylation products of these primary cannabinoids is shown in the Supplementary Figure S3 and follows the same trends seen in Figure 2, albeit in much lower amounts. The level of Δ^9^-THC was lower while the level of CBG was considerably higher in CP-outdoor samples. Δ^9^-THC is the primary psychotropic metabolite of cannabis and binds to specific G-protein-coupled receptors, cannabinoid CB1 and CB2 receptors [31]. Although there is growing research on the potential value of THC in the treatment of a number of human diseases [26,32], its development as a therapeutic has been limited due to its psychoactive properties. However, CBD, CBG, and CBC have very low affinity for CB1/CB2 receptors and have less psychotropic activities compared to THC. We detected a significantly higher level of CBD in the RV-outdoor samples compared to the indoor-grown RV samples. CBD is one of the most abundant cannabinoids and is well-known for its anxiolytic and antipsychotic properties [33]. It has been shown that CBD has multiple pharmacological benefits in in vitro and animal studies, which makes it a very promising therapeutic commodity in inflammation, diabetes, cancer, and neurodegenerative diseases [34,35]. It is demonstrated that CBG has a promising therapeutic potential in the treatment of inflammatory bowel disease and prostate cancer [35,36]. An early study by Mahlberg and Hemphil showed the higher level of THC in cannabis leaves grown under sunlight than the plants grown under filtered green light and darkness while there was no significant difference in THC content in plants grown under filtered blue and red lights and shaded daylight compared to the sunlight grown plants [37]. Moreover, they showed that the level of CBC was maintained comparable or lower in plants grown under daylight than light stressed conditions. CBC is particularly abundant in young plants or freshly harvested dry-type cannabis and is hypothesized to be a synergist for the psychoactive cannabinoids [26,38,39,40].

**Figure 3:**
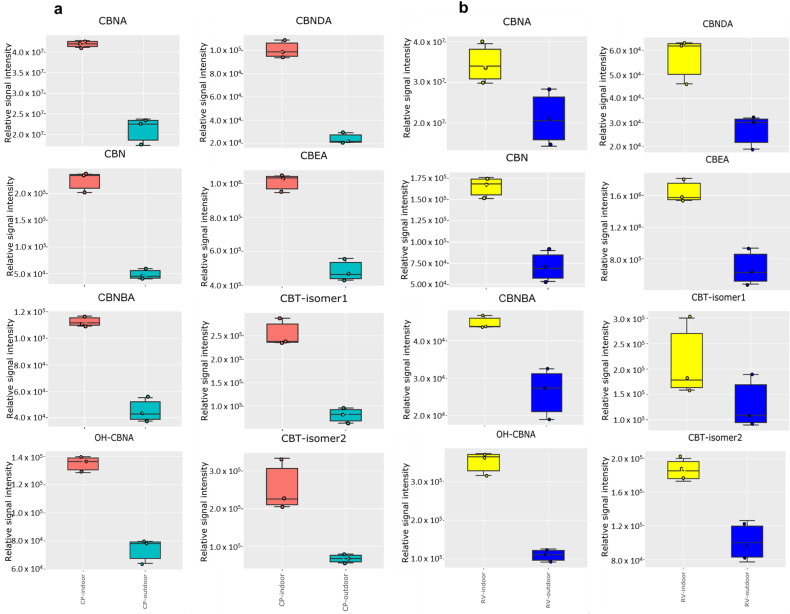
Comparison of the cannabinoids that resulted from oxidation and degradation of the cannabinoids CBCA, Δ^9^-THCA, and CBDA for (**a**) CP-indoor and CP-outdoor; (**b**) RV-indoor and RV-outdoor measured by LC-MS analysis (n = 3 independent samples per group).

A more detailed analysis of all the extracted signal intensities using volcano plots was performed to visualize independent changes in cannabinoid profile and to discriminate between outdoor and indoor-grown cultivars (Supplementary Figure S2b). The levels of 42 and 32 ion features were remarkably higher (FDR-corrected *p*-value < 0.05 and fold change threshold of 2) in the indoor-grown CP and RV groups, respectively. Moreover, the relative abundance of 42 ion features was significantly lower in the indoor-grown CP samples (Supplementary Figure S2c). By removing different co-existing adduct ions, in-source fragment ions, and finally matching the MS/MS fragments with available commercial standards or reported in literature, we could annotate 21 unique cannabinoids as shown in Table S1. Intriguingly, we found significant differences in the levels of the cannabinoids between the two cultivation methods that produced through the environment the cannabis is subject to during growth, curing, and packaging, as shown in Figure 3. For both cultivars, the oxidation and degradation products of the primary cannabinoids, including CBN, CBNA, OH-CBNA, CBNBA, CBNDA, CBEA, CBT-isomer 1, and CBT-isomer 2, and others, are significantly amplified in the indoor-grown samples. CBNDA and CBEA are the results of full aromatization and photo-oxidation of CBDA, respectively. CBT isomers are the hydroxylated forms of THC [41]. CBN and its derivatives and analogs are synthesized from the oxidative aromatization of their corresponding THC-type derivatives [34]. The continued exposure of CBN to ultraviolet light in the presence of oxygen or air produces degradation products, OH-CBN. Interestingly, we found over two orders of magnitude more CBNA compared to CBN in these samples (Figure 3), indicating that CBNA produced from THCA and consequently, CBNA becomes CBN through spontaneous or induced decarboxylation.

It should also be noted that CBNA is not currently being tested on the California Certificate of Analysis (COA) for cannabis. Therefore, consumers might be exposed to much more CBN after heating the samples than is depicted on the COA. Significant accumulation of CBNA and the plethora of other oxidation and degradation products such as OH-CBNA and CBNBA in the indoor samples is imperative because many of these oxidized cannabinoids might have diverse biological activities [42]. For example, it is shown that CBN is a strong sedative when it is combined with THC [43].

**Figure 4:**
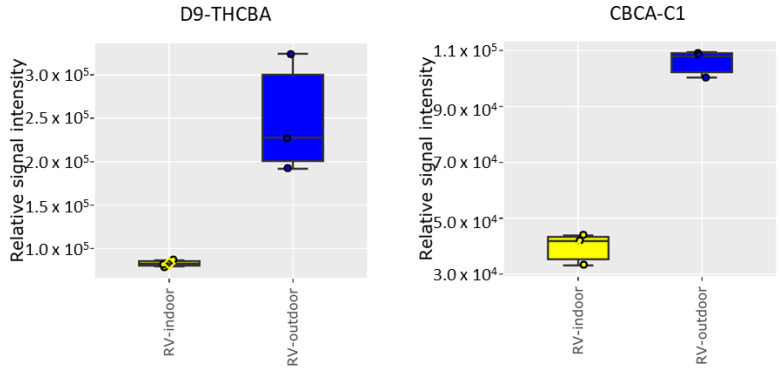
Unusual cannabinoids detected in more abundance in RV-outdoor compared to RV-indoor using LC-MS analysis (n = 3 independent samples per group).

Furthermore, we found other cannabinoids produced in much greater quantity in the outdoor-grown samples, as shown in Figure 4. This is particularly acute in the samples of RV-outdoor. We observed increased levels of Δ^9^-THCBA as well as CBCA-C1. There is also an indication that the outdoor samples may contain the C6 version of Δ^9^-THCA, but this molecular ion was difficult to validate conclusively due to its similar fragmentation patterns with THCMA compound [44] (Supplementary Figure S3). The THCA derivatives with different length hydrocarbon tails are significant because they could have suitable biological activities [45,46]. Δ^9^-THCBA possesses psychoactive properties but has been reported to have less anxiety associated with it, which is an issue with Δ^9^-THC in prescribed medicines such as Marinol (dronabinol) for treating nausea and loss of appetite associated with cancer chemotherapy and AIDS [46,47,48]. The Δ^9^-THCHA has been shown to have antinociception activity in mice [44]. Therefore, outdoor growing and breading plants to express larger quantities of these active components would be beneficial from a medicinal viewpoint.

### 2.3. Terpene Analysis

The structure and classification of terpenes are based on linking numerous isoprene units, which are mainly classified as monoterpenes and sesquiterpenes. These volatile compounds in cannabis are synthesized alongside phytocannabinoids in the glandular trichomes. Terpenes are responsible for the aroma characteristic of cannabis and have a significant role in the defense system, serving in a range of defense strategies against pests, fungi, and bacteria [49]. Moreover, the resinous content of the trichomes makes them sticky, creating a trap for insects [50]. We measured the terpene levels using targeted GC-MS analysis in both RV and CP cultivars produced either indoors or outdoors. Terpenes’ annotations were determined by matching with commercial standards and the NIST library. We identified 9 monoterpenes and 14 sesquiterpenes in CP groups and 10 monoterpenes and 15 sesquiterpenes in RV samples (Tables S2 and S3). In agreement with many previous studies on cannabis [49,51], we also detected the most commonly reported terpenes including myrcene, terpineol, limonene, α-pinene, linalool, humulene, and caryophyllene in both cultivars. Interestingly, we detected fenchone and several sesquiterpenes such as aristolene, selina-diene, trans-sesquisabinene hydrate, γ-elemene, and β-maaliene only in RV samples and β-bisabolene, alpha-bisabolene, bulnesol, and chamigrene only in CP samples, highlighting the different terpenes profile for each cultivar.

**Figure 5:**
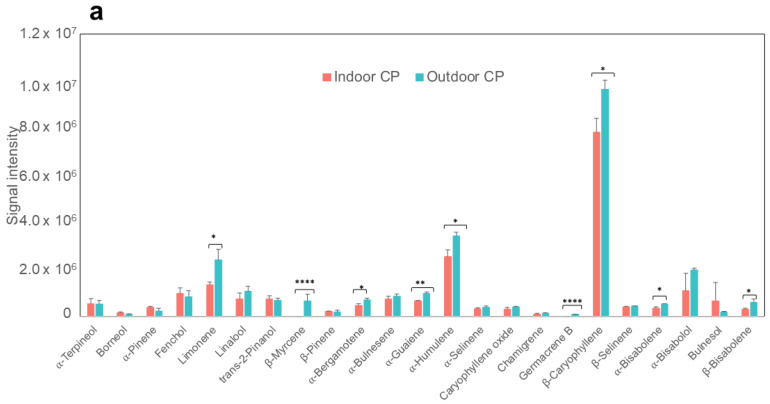

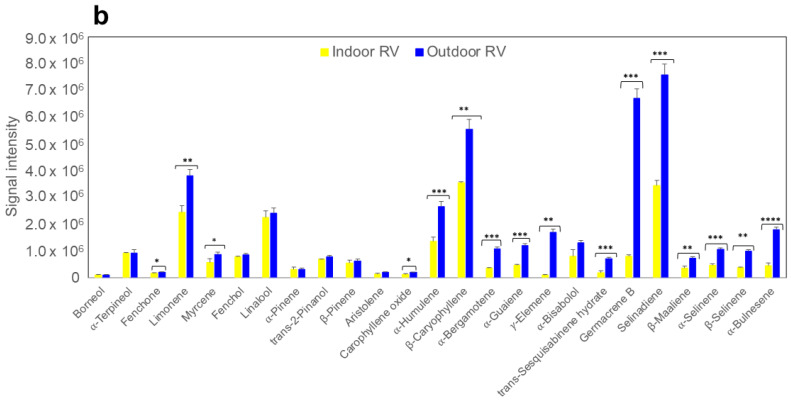
Identified terpenes from targeted GC-MS analysis in (**a**) CP-indoor and CP-outdoor, (**b**) RV-indoor and RV-outdoor. Data are plotted as the average ± SD, n = 3 individual samples; **** *p* < 0.0001, *** *p* < 0.001, ** *p* < 0.01, * *p* < 0.05 as calculated by t-test.

Among the significantly differentiated terpenes, we found remarkably higher levels of limonene, β-myrcene, β-caryophyllene, α-humulene, α-bergamotene, α-guaiene, and germacrene B in outdoor samples in both cultivars (*p*-value < 0.05) as shown in Figure 5. This is particularly acute for the samples of RV-outdoor, where the predominant terpene was a sesquiterpene selina-diene, which is not being tested on the California COA for cannabis. β-Caryophyllene (BCP) is one of the most abundant sesquiterpenes in cannabis plants and extracts, which is well known for its antimicrobial, antifungal, antioxidant, and anticarcinogenic properties [52,53]. It is shown that BCP is a strong CB2 agonist and has anti-inflammatory effects in DSS-induced colitis mouse models [53,54]. The oxidized BCP can alter cancer-related pathways, such as MAPK, STATS pathways, by induction of reactive oxygen species generation in prostate and breast cancer cell lines independent of endocannabinoid system machinery [53,55]. The signal intensity of oxidized BCP was significantly higher in RV-outdoor samples compared to indoor groups. Oral administration of α-humulene (formerly known as α-caryophyllene) in a mouse model of airways allergic inflammation can lessen eosinophilic migration into the BALF and lung tissues by reduction of inflammatory mediators NF-kB and AP1 [56]. The average signal intensity of α-bergamotene, a minor sesquiterpene, was three times higher in RV-outdoor samples compared to the indoor group. It is shown that α-bergamotene secretion by NaTPS38 terpene synthase, in wild tobacco mediates both defenses against herbivores in leaves and pollinator attraction in flowers [57]. Another enriched sesquiterpene detected in outdoor samples, especially in the RV-outdoor group is germacrene B, which is reported to have remarkable antimicrobial activity [58]. Interestingly, the CP-indoor samples lack germacrene B, which could be a reflection of the growth conditions of indoor samples. α-guaiene is a precursor to rotundone, which is an aroma compound reported in some wine verieties [59]. Limonene is a precursor compound to monoterpenoids and shows different pharmacological properties including anti-inflammatory, gastro-protective, anti-nociceptive, anti-tumor, and neuroprotective. It is also reported to be an antidote to excessive psychoactive adverse events produced by THC [26]. Remarkably, we found that the CP samples grown indoor completely lacked β-myrcene. β-myrcene is a major monoterpene and can intensify the anti-stress, anxiolytic, and sedative effects of CBD [60].

Moreover, oral administration of β myrcene in mice demonstrated remarkable effects against oxidative damage in peptic ulcers and cerebral ischemic brain injury by increasing the level of glutathione peroxidase and total glutathione in the tissues [61,62]. The main finding is that the outdoor cannabis samples had a greater diversity of terpenes and greater amounts of the ones that are present when compared to indoor cannabis from the same genetic stock. Moreover, the outdoor samples have a greater preponderance of sesquiterpenes relative to the indoor samples. Therefore, in-depth metabolomic evaluations of cannabis terpene profiles grown in different conditions are important given their potential medicinal and therapeutic values. Moreover, our results suggest that the remarkable differences in the terpene compositions may be a reflection of indoor growers not optimizing growing conditions for terpenes that do not appear in the California testing. It has been well documented that terpene levels in cannabis have been declining over the past decade or so [63,64].

It is not clear why the indoor samples produce more degraded and oxidized cannabinoids. However, this could be related to the synergism that the plant has evolved throughout its history. One of the terpenes’ functions in the plant is to act as an antioxidant and can also protect the plants for pest damage [65,66]. When grown indoors in the controlled environment, we found that the terpenes are not expressed in as high an amount. Therefore, there is less of an oxidation shield provided to the flowers in indoor cannabis. This could account for the increased levels of oxidized and degraded cannabinoids in indoor samples. For example, the sesquiterpenes and cannabinoids are produced on different biochemical pathways and we found that the several metabolites related to the sesquiterpene pathway are accessed more effectively in outdoor cultivation. In parallel with this, the outdoor plants are able to express the totality of their biochemical pathways. Terpenes can act synergistically with variations in quantities and ratios and in combinations with other bioactive secondary metabolites such as annabinoids as suggested by the varied medicinal efefcts, known as the “entourage effect” [26]. This synergy could be significant in cultivating and breeding cannabis with greater therapeutic benefits.

## 3. Materials and Methods

### 3.1. Chemicals and Reagents

Optima LC-MS grade acetonitrile, formic acid, methanol, water, and HPLC grade ethanol (Absolute, 200 proof, molecular biology grade) were purchased from Fisher Scientific (Hampton, NH, USA). The phytocannabinoid analytical standards Δ9-Tetrahydrocannabinolic acid (THCA), Δ^9^-Tetrahydrocannabinol (THC), Δ9-Tetrahydrocannabivarin (THCV), Δ^8^-THC, Cannabigerolic acid (CBGA), Cannabigerol (CBG), Cannabinolic acid (CBNA), Cannabinol (CBN), Cannabichromene (CBC), Cannabidiolic acid (CBDA), Cannabidiol (CBD), Cannabidivarinic acid (CBDVA), and Cannabicyclol (CBL) were purchased from SigmaAldrich (St. Louis, MA, USA).The following terpene standards were purchased from Toronto Research Chemicals (North York, ON, Canada): Carvone, Ledene, Viridiflorol, β-Guaiene, trans-β-Farnesene, β-Bisabolene, β-Elemene, α-Eudesmol, β-Eudesmol, γ-Eudesmol, β-Sesquiphellandrene, α-Zingiberene, α-Selinine, β-Selinene, Bulnesol, Aromadendrene, Sabinene, Hydrate Sabinene, and α-Thujene. The following terpene standards were purchased from: Milli-pore Sigma (Burlington, MA, USA): Longifolene solution, cis/trans-Ocimene, Guaiol, Terpinolene, Valencene, Caryophyllenne oxide. The following standard mixtures were purchased from Sigma Aldrich: Cannabis Terpene Mix A which includes β-Pinene, Camphene, α-Pinene, 3-Carene, α-Terpinene, Limonene, gamma-Terpinene, L-Fenchone, Fenchol, Camphor, Isoborneol, Menthol, Citronel-lol, Pulegone, Geranyl acetate, α-Cedrene, α-Humulene, Nerolidol, Cedrol, α-Bisabolol, and Cannabis Terpene Mix B which includes β-Pinene, β-Caryophyllene, Phytol, Limonene, Geraniol, Camphor, Terpinolene, β-Eudesmol, Borneol, cis-Nerolidol, α-Terpineol, Carene, Linalool, and p-Cymene.

### 3.2. Sample Preparation

The use of plants in the present study complies with international, national, and institutional guidelines. Cannabis plants were purchased from commercial suppliers and as such, they were compliant with the state of California guidelines. We chose flowers from the upper third of the plants with similar morphology and size to standardize the sampling. Three independent plant samples were prepared from each sample to address variance in the samples.

Two commercial cultivars of cannabis were analyzed, Red Velvet (RV, Batch #: 210727-1LDX-GEN-RVT) and Cheetah Piss (CP, Bach #210524-1LDX-SJM-CPIS). Each of the samples was from the same season to eliminate issues of large differences in age between the samples. The outdoor samples were part of 2021 seasonal and commercial grow by Ridgeline Farms. They are referred to here as RV-outdoor and CP-outdoor. The indoor samples were grown commercially in 2021 from clones of the same genetic stock as the outdoor samples. The indoor samples were grown by grandifloragenetics.com for RV and by cookies.com for the CP samples. These samples are referred to here as RV-indoor and CP-indoor. The outdoor samples were grown in raised beds using a proprietary mixture of all-natural soil and composts under full sunlight. The indoor samples were grown under artificial light in a proprietary growth medium. Samples of the trimmed and cured cannabis flowers (late flowering phase) were prepared as described previously [42]. Briefly, triplicates of manually ground flowers (250 mg) were weighed into glass vials and extracted with ice-cold ethanol. These extracted samples were filtered twice through 0.44 µm PTFE filters. These solutions were then utilized for both the GC-MS evaluation of the terpene composition or UPLC-MS evaluation of the cannabinoids.

### 3.3. LC-MS Analysis

For the analysis of the cannabinoids, aliquots of the stock solutions diluted 10 and 100 times with ethanol and injected in duplicates in randomized orders onto the LC-MS for analysis. Chromatographic separation was performed on Acquity UPLC H-Class system (Waters Corporation, Milford, MA, USA) using a Kinetex C18 core-shell column (2.6 μm, 100 mm × 2.1 mm) and a ternary multistep gradient. Mobile phase A was consisted of water and mobile phase B consisted of acetonitrile both containing 0.1% formic acid, and mobile phase C included methanol and kept constant at 5% throughout the run. The chromatographic gradient was set as follows: 0–1.3 min 45% A and 50% B, 1.3–2.67 min 28% A and 67% B, 2.67–6.67 min 5% A and 90% B, 6.67–9.33 90% B, 9.33–10 min 45% A and 50% B, 10–14 min 45% A and 50% B. The flow rate was set to 0.3 mL/min and the column temperature was set at 30 °C. The UPLC was coupled to a Xevo G2 XS Q-ToF MS (Waters Corporation, Milford, MA, USA), and operated in both positive and negative electrospray ionization modes. The capillary voltage and sampling cone voltage of 2 kV and 32 V were used in the positive mode. The source and desolvation temperatures were 120 °C and 500 °C, respectively. The desolvation gas flow (N2) was set to 650 L/hr. For the negative mode, a capillary voltage of −1.5 kV and a cone voltage of 30 V were used. Accurate mass was obtained by injections of leucine enkephalin as a lock spray. The data was collected in duplicates over the mass range *m*/*z* 50 to 700 Da. Quality control (QC) from a pooled aliquot of samples was injected at the beginning, between the samples, and at the end of the runs in order to monitor for retention time drift and the stability of the MS platform. The QC samples were also acquired in both MS/MS and data-independent MSE mode for the structural assignment of the cannabinoids. The low collision energy was set to 4 eV, and the trap collision energy was ramping from 20 to 45 eV.

### 3.4. GC-MS Analysis

For the analysis of the terpenes, 2 µL of samples described above were subjected to Agilent 7890B/5977B GC-MS system. The samples were analyzed in splitless mode with a DB-5MS capillary column (30 m × 0.25 mm × 0.25 µm; Agilent, J & W Scientific, Santa Clara, CA, USA). High purity helium was used as a carrier at a flow rate of 1.0 mL/min. The injector and ion source temperatures were set at 280 °C, and 230 °C, respectively. The temperature program was conducted as follows: the initial temperature was 35 °C for 2 min; then the temperature was increased to 150 °C at a rate of 15 °C/min, and maintained for 5 min at this temperature; next, the temperature was increased to 290 °C at a rate of 3 °C/min; and finally, the temperature at 290 °C was held for 2 min. The mass spectra were acquired with electron ionization mode at 7 eV in full scan mode (*m*/*z* 60–500 Da).

### 3.5. Data Pre-Processing and Statistical Analyses

The LC-MS raw data files were converted to netCDF format using DataBridge tool implemented in MassLynx software (Waters, Version 4.1). Then, they were subjected to peak-picking, retention time alignment, and grouping using XCMS package in R (Version 3.5.1) environment as described previously64. The output data frame included a list of time aligned detected features (*m*/*z*, retention time) and the relative signal intensity in each sample. Multivariate and univariate statistical analysis were performed in MetaboAnlyst 5.0 and also in R environment. PCA analysis was performed on auto-scaled and log-transformed data. Group differences were calculated using Welch *t*-test with FDR corrected *p*-value < 0.05. The fold change (FC) in each metabolite abundance was calculated by comparing the mean values of the peak areas in each group. The Volcano plot was constructed by plotting the log_2_ FC (outdoor/indoor) of extracted features against log_10_ *p*-value. The GC-MS data were processed in MNOVA. Given the multitude of terpenes, terpenoids, sesquiterpenes, and sesquiterpenoids in these samples some of them were identified with the aid of the NIST database. The retention index and mass similarity were considered in terpene assignments. The utility of the identified terpenes and cannabinoids as potential predictive markers to distinguish outdoor-grown cultivars from indoor-grown ones was calculated using a multivariate receive operating characteristic (ROC) curve in MetboAnalyst 5.0.

## 4. Conclusions

Our complementary targeted GC-MS and untargeted LC-MS analyses showed significant differences in the terpene and cannabinoid profiles of two cultivars of cannabis grown in two different conditions. One important conclusion of this study is that the consumer is not being given a complete picture of the components in cannabis. Numerous oxidized and degraded cannabinoids are present, and many of them may have adverse or unknown biological indications. Whether the cannabis is grown indoors under artificial lights using artificial growth media or outdoors in living soil with sunlight influences the types and amounts of molecules that are present in the flowers. Indoor samples have a greater preponderance of oxidized and degraded cannabinoids, and the outdoor samples are able to express more cannabinoids with potentially desirable bioactivity. Therefore, a comprehensive understanding of the composition of secondary metabolites, such as cannabinoids and terpenes grown in different environmental conditions, is of primary importance for the medical and recreational use of cannabis. Growing cannabis that expresses the unusual cannabinoids, such as C4- and C6-Δ^9^-THCA, could have significant medicinal benefit. There is also an important conclusion from this study revealing inadequacies in California COA testing to delineate important components of cannabis that are being sold. The lack of testing for many of the important terpenes (e.g., sesquiterpenes), cannabinoids (e.g., THCA derivatives with different length hydrocarbon sidechains), and their degradation products (e.g., CBNA) highlights the deficiency of the California COA testing. The ROC curve analysis using a random forest model revealed that α-guaiene, α-bergamotene, CBN, CBNDA, and CBT could serve as the top 5 potential predictive markers (AUC = 0.995) for these cultivars to discriminate the outdoor-grown from indoor ones. This study is the first to evaluate the impact of natural and artificial cultivations on the profile of cannabinoids and terpenes in commercial cannabis. However, our analysis was limited by the restricted samples sizes and the limited information on the growing conditions of each cultivar in each environment. Further studies with larger sample sizes and different environmental conditions and breeds could enhance our understanding of the bio-chemical diversities of cannabinoids and terpenes with different medicinal and physiological properties.

## Data Availability

The datasets generated during and/or analyzed during the current study are available from the corresponding author upon reasonable request.

## Supplementary Materials

The following supporting information can be downloaded at: https://www.mdpi.com/article/10.3390/molecules28020833/s1, Figure S1. Representative total ion chromatograms of untargeted LC-MS analysis (**a**) and PCA score plots (**b**) of the extracted metabolic features from all the groups in both negative and positive electrospray ionization modes. CP (Cheetah Piss; n = 3 independent sample), RV (Red Velvet; n = 3 independent sample), QC (Quality Control; n = 6). Figure S2. The loadings and volcano plots derived from extracted metabolic features from untargeted LC-MS analysis in the indoor-grown as compared with the outdoor-grown CP (**a**,**b**) and RV samples (**c**,**d**). The volcano plot highlights the significantly differentiated metabolic features that increased (shown in red, Sig-Up) or decreased (shown in blue, Sig-Down) with the fold change threshold of 2 and FDR-corrected p-value < 0.05. Figure S3. Comparison of the common decarboxylated cannabinoids, CBG, CBC, Δ9-THC, and CBD detected by LC-MS analysis in the CP (**a**) and RV (**b**) samples (n = 3 independent samples per group). Figure S4. The level of tentatively annotated d9-THCHA in RV samples (n = 3 independent sample per group). Table S1. The list of annotated cannabinoids from untargeted LC-MS/MS analysis from outdoor- versus indoor- grown RV and CP samples. Fold change values calculated from average signal intensity of outdoor samples to average signal intensity of indoor samples (n = 3 independent sample per group). Δppm = mass error. Table S2. Results from t-test analysis of the detected terpenes by targeted GC-MS analysis from outdoor-versus indoor-grown CP samples. Fold change values calculated from average signal intensity of outdoor samples to average signal intensity of indoor CP samples (n = 3 independent sample per group). Table S3. Results from t-test analysis of the detected terpenes by targeted GC-MS analysis from outdoor-versus indoor-grown RV samples. Fold change values calculated from average signal intensity of outdoor samples to average signal intensity of indoor RV samples (n = 3 independent sample per group).

## Sample Availability

Data of evaluated cannabis extracts are available from the authors.
