# Supplementary material for: Comparison of the Cannabinoid and Terpene Profiles in Commercial Cannabis from Natural and Artificial Cultivation
Source: Molecules. 2023 Jan 13;28(2):833. doi: 10.3390/molecules28020833 (PMC9861703; doi:10.3390/molecules28020833)
Supplement: Supplementary file 1 [file molecules-28-00833-s001.zip › molecules-2133011-supplementary.pdf]

Supplementary Materials

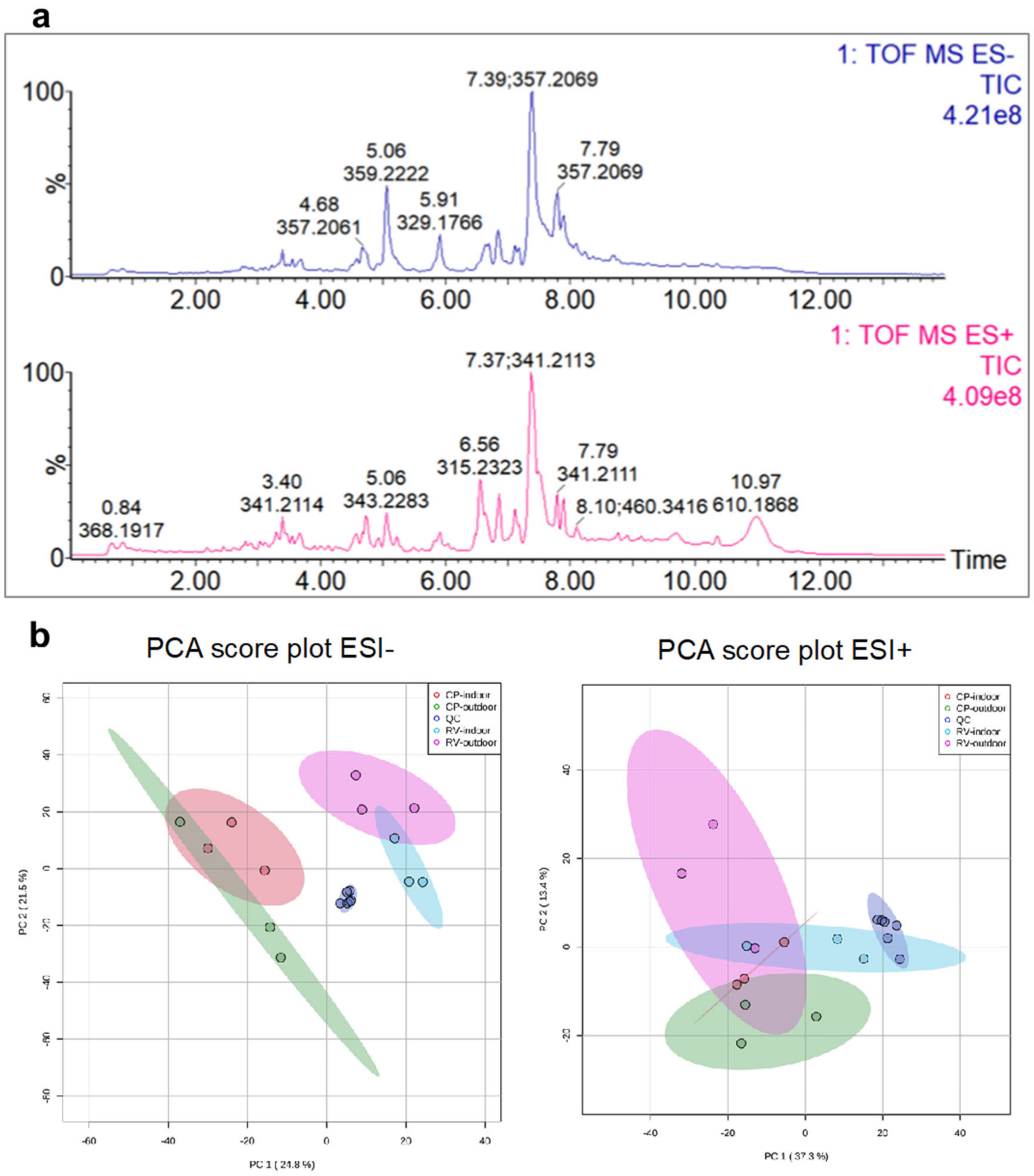

Figure S1. Representative total ion chromatograms of untargeted LC-MS analysis (**a**) and PCA score plots (**b**) of the extracted metabolic features from all the groups in both negative and positive electrospray ionization modes. CP (Cheetah Piss; n=3 independent sample); RV (Red Velvet; n=3 independent sample), QC (Quality Control; n=6).

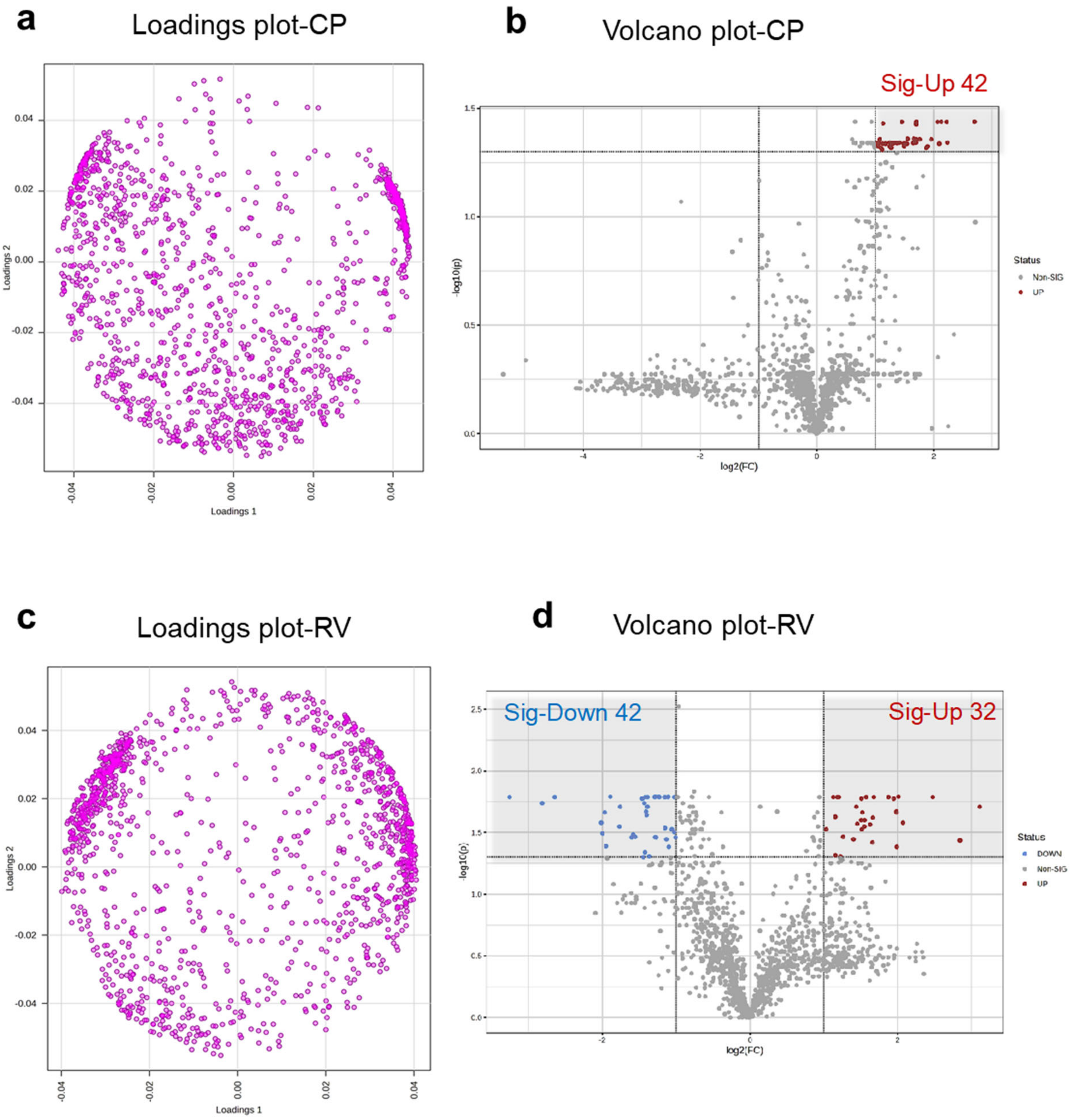

Figure S2. The loadings and volcano plots derived from extracted metabolic features from untargeted LC-MS analysis in the indoor-grown as compared with the outdoor-grown CP (**a and b**) and RV samples (**c and d**). The volcano plot highlights the significantly differentiated metabolic features that increased (shown in red, Sig-Up) or decreased (shown in blue, Sig-Down) with the fold change threshold of 2 and FDR-corrected p-value < 0.05.

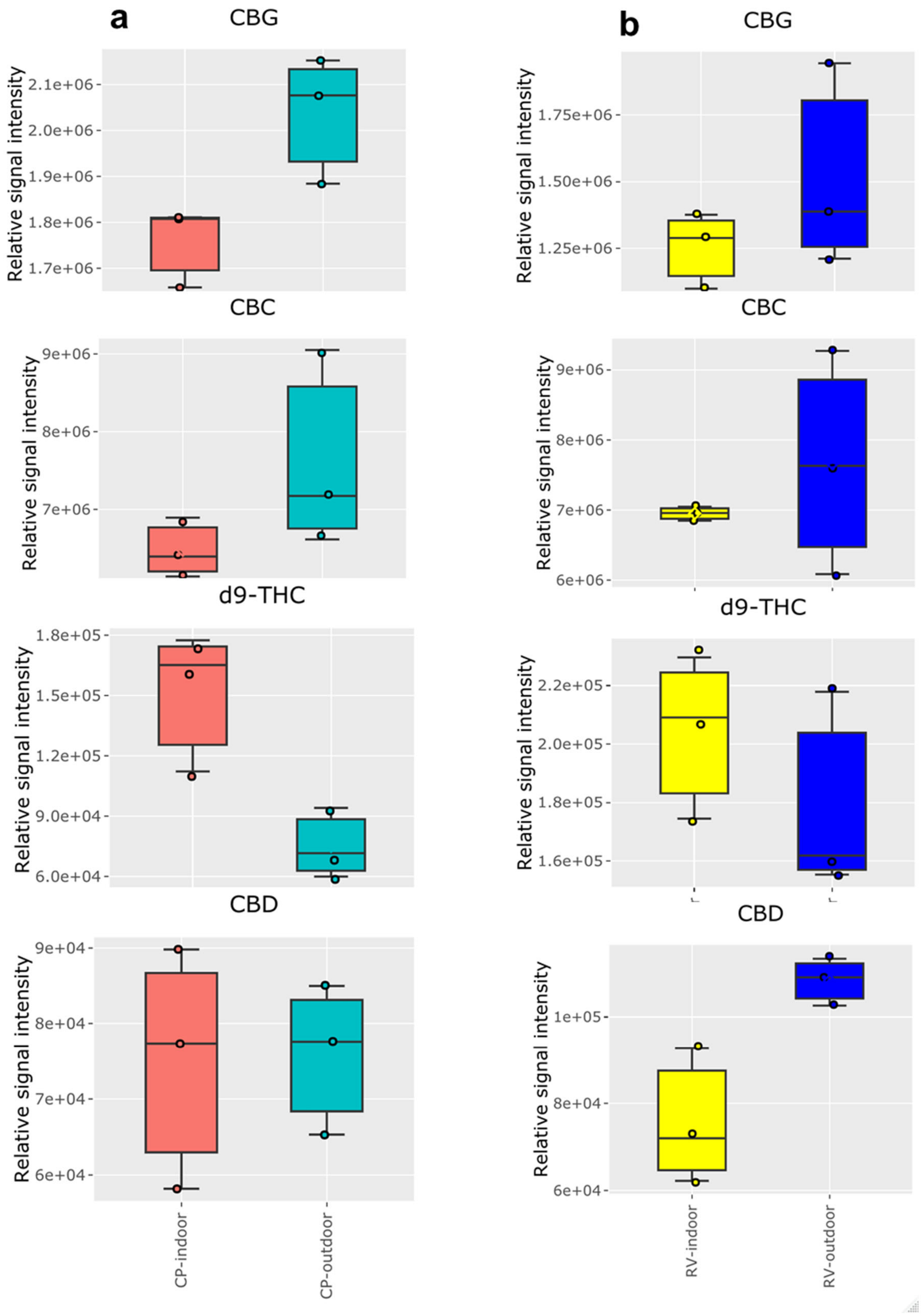

Figure S3. Comparison of the common decarboxylated cannabinoids, CBG, CBC, Δ^9-THC, and CBD detected by LC-MS analysis in the CP(**a**) and RV (**b**) samples (n=3 independent samples per group).

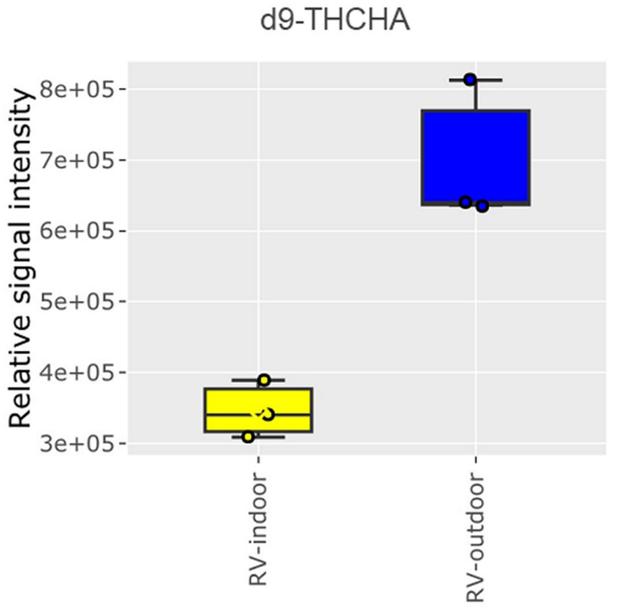

Figure S4. The level of tentatively annotated d9-THCHA in RV samples (n=3 independent sample per group).

Table S1. The list of annotated cannabinoids from untargeted LC-MS/MS analysis from outdoor- versus indoor- grown RV and CP samples.
Fold change (FC) calculated from average signal intensity of outdoor samples to average signal intensity of indoor samples (n=3 independent sample per group).
Δppm = mass error.

Name	M/z detecetd as [M-H]	Retention Time	Δppm	Major Fragments	FDR-corrected P-value	FC RV-Outdoor/-Indoor	FDR-corrected P-value	FC CP-Outdoor/Indoor
CBGA	359.222	5.05	0.56	341.2120, 315.2328, 191.1082	0.73	0.93	0.57	1.11
CBCA	357.2066	7.76	0.28	313.2140, 261.1107, 243.1339, 191.1026	0.02	1.89	0.66	1.07
d9-THCA	357.2067	7.39	0.28	313.2103, 245.1502, 191.1026	0.15	0.64	0.66	0.90
CBDA	357.2068	4.65	0.56	339.1983, 245.1566, 179.1094	0.46	1.31	0.82	1.03
CBG	315.2321	5.03	0.95	245.1566, 191.1082, 179.1094	0.46	1.21	0.28	1.16
CBD	313.2163	4.65	1.6	245.1502, 179.1066	0.15	1.43	0.95	1.01
CBC	313.2168	7.37	0.32	243.1403, 203.1076, 191.1082, 179.10666	0.65	1.10	0.54	1.18
d9-THC	313.2164	6.53	1.28	245.1566, 191.1082	0.48	0.87	0.19	0.50
CBNA	353.1753	6.82	0.28	309.1886, 279.1396, 222.0708, 171.0814	0.21	0.61	0.04	0.50
CBN	309.1848	5.96	2.26	279.1327, 222.0647, 171.0814	0.05	0.43	0.02	0.21
CBNBA	339.1592	6.05	1.18	295.1286, 265.0872	0.12	0.59	0.03	0.40
OH-CBNA	369.1695	3.82	1.9	325.1807, 295.1240, 187.051	0.01	0.31	0.03	0.55
CBNDA	353.175	3.67	0.85	309.1454, 279.132, 252.083, 171.0815	0.09	0.47	0.02	0.24
CBEA	373.2014	3.35	1.88	311.1584, 205.0965	0.07	0.41	0.03	0.48
CBT-iso1	345.206	3.05	1.74	327.1487, 309.1418, 285.1467, 191.0815, 179.0834	0.33	0.61	0.03	0.32
CBT-iso2	345.2061	3.73	1.45	327.1525, 309.1454, 285.1467, 191.0815, 179.0861	0.08	0.54	0.04	0.27
d9-THCBA	343.1905	7.09	1.17	325.1364, 299.1610, 177.0661	0.03	2.99	0.78	0.93
CBCA-C1	301.1434	5.08	1.99	257.1172, 283.0957, 187.0511, 135.0452	0.01	2.67	0.74	0.80
d9-THCHA	371.2218	7.74	1.08	327.2402, 205.1276, 205.1276, 191.1108	0.03	2.01	0.92	1.01
CBDVA	329.1749	3.55	1.22	311.1619, 217.1201, 163.0753, 151.0732	0.72	1.15	0.49	0.70
d9-THCVA	329.1755	5.86	0.61	285.1884, 217.1232, 163.0753	0.67	1.12	0.52	0.61

Table S2. Results from t-test analysis of the detected terpenes by targeted GC-MS analysis from outdoor- versus indoor-grown CP samples.

Fold change values calculated from average signal intensity of outdoor samples to average signal intensity of indoor CP samples (n=3 independent sample per group).

Name	Chemical Class	P-value	Fold change
Borneol	monoterpene	0.6991	0.95
α-Terpineol	monoterpene	0.8766	1.01
Fenchone	monoterpene	0.0328	1.22
Limonene	monoterpene	0.0022	1.56
Myrcene	monoterpene	0.0493	1.51
Fenchol	monoterpenoid	0.2011	1.07
Linalool	monoterpenoids	0.4679	1.06
α-Pinene	monoterpens	0.9211	0.98
trans-2-Pinanol	monoterpens	0.1625	1.11
β-Pinene	monoterpens	0.3823	1.12
Aristolene	Sequiterpenes	0.0672	1.54
Carophyllene oxide	Sequiterpenes	0.0242	1.75
α-Humulene	Sequiterpenes	0.0013	1.95
β-Caryophyllene	Sequiterpenes	0.0110	1.56
α-Bergamotene	Sesquiterpene	0.0006	3.07
α-Guaiene	sesquiterpene	0.0008	2.54
γ-Elemene	sesquiterpene	0.0017	16.09
α-Bisabolol	sesquiterpene	0.0553	1.63
trans-Sesquisabinene-hydrate	sesquiterpenes	0.0009	3.93
Germacrene B	Sesquiterpenes	0.0009	8.44
Selinadiene	Sesquiterpenes	0.0008	2.20
β-Maaliene	Sesquiterpenes	0.0024	2.02
α-Selinene	sesquiterpenoid	0.0003	2.30
β-Selinene	sesquiterpenoid	0.0017	2.58
α-Bulnesene	Sesquiterpenoids	0.0001	3.95

Table S3. Results from t-test analysis of the detected terpenes by targeted GC-MS analysis from outdoor- versus indoor-grown RV samples.
Fold change values calculated from average signal intensity of outdoor samples to average signal intensity of indoor RV samples (n=3 independent sample per group).

**Name**	**Chemical Class**	**P-value**	**Fold change**
α-Terpineol	Monoterpenoid	0.9194	0.97
Borneol	Monoterpenoid	0.1289	0.69
α-Pinene	Monoterpene	0.1466	0.62
Fenchol	Monoterpene	0.5277	0.86
Limonene	Monoterpene	0.0432	1.78
Linalool	Monoterpene	0.1648	1.43
trans-2-Pinanol	Monoterpene	0.5619	0.92
β-Myrcene	Monoterpene	NA	NA
β-Pinene	Monoterpene	0.7736	0.94
α-Bergamotene	Sequiterpenes	0.0198	1.50
α-Bulnesene	Sequiterpenes	0.2995	1.15
α-Guaiene	Sequiterpenes	0.0042	1.49
α-Humulene	Sequiterpenes	0.0211	1.35
α-Selinene	Sequiterpenes	0.3156	1.17
Caryophyllene oxide	Sequiterpenes	0.1594	1.30
Chamigrene	Sequiterpenes	0.2413	1.22
Germacrene B	Sequiterpenes	NA	NA
β-Caryophyllene	Sequiterpenes	0.0153	1.23
β-Selinene	Sequiterpenes	0.0638	1.10
α-Bisabolene	Sesquiterpens	0.0245	1.46
α-Bisabolol	Sesquiterpens	0.1743	1.79
Bulnesol	Sesquiterpens	0.4146	0.30
β-Bisabolene	Sesquiterpens	0.0509	1.87
